# Concussion injury management, perception, and knowledge in amateur field hockey

**DOI:** 10.17159/2078-516X/2023/v35i1a15697

**Published:** 2023-09-21

**Authors:** C King, H Morris-Eyton

**Affiliations:** Department of Sport and Movement Studies, Faculty of Health Science, University of Johannesburg, South Africa

**Keywords:** sports-related concussion, injury reporting, non-contact sport

## Abstract

**Background:**

Field hockey has a high risk for sports-related concussion (SRC) injuries due to the speed and intensity of the game, current rules, field surfaces and equipment composition. Head injuries are the second most common reported injury and up to 75% of SRCs go unreported or undetected. This increases the subsequent injury risk, long-term health consequences and prolonged injury recovery.

**Objectives:**

This study aimed to examine the prevalence of SRC in hockey players within the Southern Gauteng Hockey Association (SGHA) premier league. Concussion knowledge and attitudes of hockey players, coaches, umpires, and officials were also investigated.

**Methods:**

A partially mixed sequential dominant status design (QUANT–qual) was used, divided into two phases. In Phase One hockey players, coaches, umpires, and technical officials (n=119) completed a modified RoCKAS-ST questionnaire. In Phase Two, a focus group discussion with umpires (n=3) and interviews with coaches (n=3) were conducted.

**Results:**

Injuries to the shoulder, neck, head, and face were reported from stick use (n=98); ball use (n=102) and collisions (n=187). Only 19% of hockey players were diagnosed with SRC, indicating that many of these injuries were undetected or not reported. Responses from the focus group discussion and interviews indicated that coaches, umpires, and officials felt they had insufficient knowledge of SRC.

**Conclusion:**

The recognition and management of on-field injuries require improvement to enhance the injury detection system.

Field hockey (hockey) is classified as a non-contact sport; however, most injuries reported are as a result of contact.^[1]^ Modifications to rules, playing surface, and equipment have contributed to a fast paced game, elevating the risk of a concussive type of injury.^[2, 3]^ Head injuries are the second most common type of injury in hockey, after lower limb injuries, and reported SRC incidence ranges from 0–25%.^[4]^ The most common SRC mechanisms of injury in hockey are from player-to-equipment contact, with 37% of SRCs from ball-to-player contact and 24% from stick-to-player contact. ^[5]^ At an elite level, 40% of injuries to women and 27% of injuries to men were to the head and face. ^[3]^

Sports-related concussion is recognised as a serious medical condition and has been classified as a mild traumatic brain injury (mTBI). ^[6]^ In the United States of America, an estimated 1.6–3.8 million sports-related mTBI occur annually. ^[7]^ These figures may be 10 times underestimated due to athletes not recognising SRC symptoms or not reporting these injuries. ^[7]^ Sports-related concussion can be referred to as an invisible injury when compared to obvious orthopaedic injuries that occur during sports competitions. The high prevalence (50–75%) of under-reporting is primarily due to the difficulty in detecting a SRC.^[8, 9]^ Other reasons for not reporting these injuries include athletes thinking that the injury is not serious, and a lack of knowledge regarding SRC symptoms. ^[9,10, 11]^ Male athletes are less likely to report a SRC compared to females, as reporting an injury has been shown to impact on their athletic identity, and they fear being perceived as weak by their coach, team-mates, or parents.^[11, 12]^ Greater education for athletes, coaches and officials is required to elucidate the seriousness of SRC to overcome the stigma related to reporting these types of injuries. ^[11, 12]^ A disconnect exists as SRC knowledge is adequate; however, the recognition and reporting of SRC is poor. ^[11, 12, 13]^ The aims of this study were to: (1) describe the prevalence of SRC in male and female hockey players within the Southern Gauteng Hockey Association (SGHA), (2) to investigate the knowledge and attitudes of hockey players, coaches, umpires, and technical officials regarding SRC.

In South Africa, high-school hockey players had an average of 1.3 SRCs per player per career.^5^ In female university athletes, 53% of reported SRC injuries were in hockey players. ^[14]^ Research regarding the prevalence of SRC in hockey has primarily examined the incidence in small samples of elite athletes. There is a need for further investigation at the amateur level where a large percentage of players participate. ^[5]^

## Methods

### Study design and participants

This study used a partially mixed sequential dominant status design (QUANT– qual) consisting of two phases. Phase One included the completion of an online questionnaire which was distributed to nine men’s and 10 women’s teams (n=101 players), coaches (n=4), umpires (n=13), and a technical official (n=1). All were participants in the SGHA premier league between March 2018 and March 2022. Participants were recruited via convenience sampling administered through premier league hockey clubs and coaches that granted permission for data to be collected. Participants were included if they were older than 18, had played, coached or officiated in the premier league during the designated time period. Phase Two consisted of a focus group discussion with umpires and technical officials (n=3) and three individual interviews with coaches (n=3) via Google Meet over four separate days. Convenience sampling based on the participants who had already completed the online questionnaire was used to select participants for the focus group discussion and interviews. The mixing of the data occurred during the analysis to obtain an in depth understanding of the knowledge and perceptions of SRC in hockey. Participants were over the age of 18 and provided informed consent for both phases of data collection.

### Ethical considerations

This research was approved by the institution’s Research Ethics Committee (REC-643-2020). Principles of autonomy, beneficence, non-maleficence, and justice were applied and adhered to throughout the research process.

### Data collection

Two separate Google Forms questionnaires were electronically mailed to all premier league registered hockey players (n=285) and coaches (n= 29), umpires (n=30) and officials (n=15). Players (n=101) completed a separate questionnaire to umpires (n=13), coaches (n=4) and a technical official (n=1). Both sets of questionnaires gathered demographic information including age, gender, and the hockey experience levels of participants. The players were asked to self-report on injuries experienced during the four-year period between March 2018 and March 2022. After completing the demographic information, coaches, umpires and technical officials only answered questions relating to their specific designation, experiences and SRCs that they may have witnessed. Data regarding first aid training, and whether they had witnessed any SRC injuries during the study period were also gathered. Both questionnaires included the modified RoCKAS-ST (2009) questionnaire to measure attitudes and knowledge regarding concussion. ^[15]^ The modified RoCKAS-ST questionnaire comprised of five sections divided into a Concussion Attitude Index (CAI) and a Concussion Knowledge Index (CKI), which were adapted for a South African context. The internal consistency of the CAI has been established at α = 0.76. ^[15]^ Test-retest reliability of the CAI and CKI have significant positive correlations, indicating adequate levels of reliability. The content validity of the CAI has been established as an adequate measure of attitudes. Overall, the CAI is a stable measure of concussion attitudes and the CKI is an adequate measure of concussion knowledge. ^[15]^ Responses from the questionnaires were anonymous.

### Statistical analysis

Quantitative data was analysed by a statistician using IBM SPSS software (V. 27) to determine frequencies and descriptives on scales and factors. An exploratory factor analysis was conducted where empirical and theoretical reliabilities were tested. Reports of participants’ self-reported injury history were generated to show distribution and trends regarding the prevalence of SRC in hockey (*p=<0.05*). Qualitative data was recorded and transcribed for narrative analysis using ATLAS.ti (V. 22.0.6). Non-independent coders were used to generate codes and develop themes extracted from the transcribed data. The questionnaires, focus group discussions and interviews were used to gather in depth data to extrapolate trends regarding concussion knowledge and attitudes regarding SRC from different perspectives.

## Results

The demographic data of the participants is presented in Table 1. Out of the 101 hockey players, 40% identified as female (n=40), while 60% identified as male (n=61). For the coaches, umpires, and technical officials, only 11% identified as female (n=2), while the majority, 89%, identified as male (n=16).

The age range for coaches, umpires and technical officials was 27–61 years, whereas for players, it was 18–43 years. Most coaches, umpires and technical officials reported witnessing 1–5 SRC injuries (Table 2). Participants identifying as female reported higher incidences (28%) of SRC compared to those identifying as male, but had lower reporting prior to March 2018 (Table 3).

**Fig. 1:**
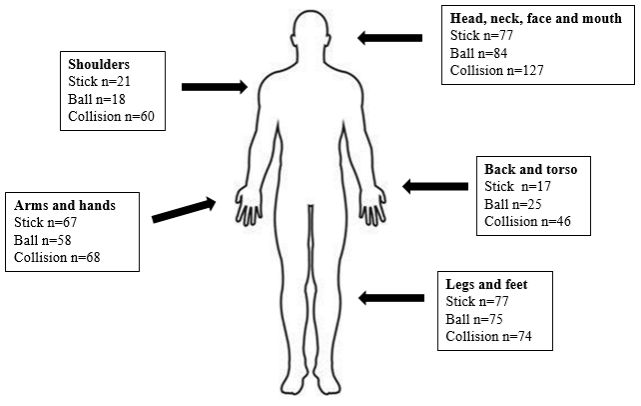
Injuries reported by players from stick use, ball use and collisions

Many injury incidents (Figure 1) occurred to the head, face, mouth, neck, and shoulders (n=387) of the players but the actual number of SRCs diagnosed following these injuries were minimal (n=20). Most coaches and officials (89%) were able to identify SRC as a head injury or identified signs and symptoms of SRC. Responses from the coaches and officials included: *‘Brain injury, resulting from impact. Recovery can be between days and months depending on severity. Usually resulting in temporary impairment of function’*. Another response was: *‘When there is a collision of players or with a ball or stick that leads to a temporary state of disorientation...It is normally caused by the swelling of the brain’*. Based on the responses, coaches and officials understood what a SRC is.

Despite being injured, 40% of players with a SRC continued to participate. This was primarily due to the severity of the injury not being recognised or that no symptoms were felt at the time of injury. Players’ responses were grouped into categories (need to win, not aware of injury severity or cleared at the time). Responses included: ‘*Did not have symptoms right away’* and *‘I didn’t realise the injury was so severe, only realised after the match.’* Another reason for continuing to play with a SRC included a need to win: *‘It was a very important game, and I didn’t realise that I was concussed at the time’* In a few cases, the athlete was checked by medical staff and cleared to continue. These results indicate a shortcoming regarding SRC knowledge, recognition, and reporting structures.

Principal Axis Factoring (PAF) revealed the presence of three items with eigenvalues above one explaining 38.6%, 15% and 11.1% of the variance respectively. The three factors were grouped and named according to common themes based on the original groupings used by Rosenbaum and Arnett ^[15]^ Empirical reliability was tested for in the three factors that resulted from the exploratory factor analysis (Table 4).

Results from the modified RoCKAS-ST (2009) questionnaire indicated an above average knowledge and attitude regarding concussion (Table 5).

All coaches, umpires, and officials who participated in phase two witnessed severe injuries: ‘*I’ve…seen people knocked out…I’ve seen broken ankles*’ (p 2); ‘*The worst would be a ball in the mouth with a significant number of broken teeth*’ (p 3); ‘*The player got hit in the throat stood back…had three mini strokes…I’ve seen a goalkeeper concussed, they got hit so hard with the ball and the helmet that they passed out’* (p 1).

## Discussion

### Prevalence of sports related concussion in male and female hockey players

Between March 2018 and March 2022, 19% of players were diagnosed with a SRC related to a hockey injury. All participants from phase two witnessed serious injuries while either coaching, umpiring, or officiating. More players identifying as female (28%) experienced a SRC compared to players identifying as male (13%), which is consistent with findings from previous studies. ^[2]^ In this study, injuries most commonly occurred from contact with a hockey ball or stick and is supported by the literature. ^[1,3,4]^ The results may have been affected by players underreporting due to the stigma related to athletic identity, survey fatigue, and decreased hockey activity due to COVID-19 lockdowns during the study period. The rates are low when compared to the number of injury incidents and is consistent with reported rates of 50–75% of SRCs that go unreported in other sports. ^[9,16]^ Injury reporting structures need to be improved in order to increase recognition and improve on-field management of injuries. For a non-contact sport numerous injuries occur from collisions or contact with a hockey stick or ball.

### Knowledge and attitudes of hockey players towards concussion

Responses from the CKI and CAI suggest that players’ concussion knowledge and concussion attitudes were above average. However, 40% of players who had a SRC continued to play whilst injured. The reasons included not recognising the injury severity, delayed symptom presentation, a need to win due to the importance of the game, or a desire to play related to athletic identity. ^[9,10, 11, 12, 13]^ Considering the serious nature of SRC injuries, education for players needs to be improved. Players were asked to explain a SRC. Answers were grouped into three categories based on the responses: 1) mechanism of injury, 2) signs and symptoms, and 3) the inclusion of loss of consciousness in the description. Most players (56%) identified SRC as a type of brain injury: *‘A blow to the brain due to impact… whether it’s directly to the head, body or neck and causes the brain to rapidly move inside the skull.’* Players (35%) were able to identify signs and symptoms: *‘A concussion presents differently… with symptoms ranging from headaches, dizziness, tiredness (fatigue), compromised vision (difficulty tracking movement with the eyes, or ‘seeing double’), nausea and vomiting.’* Finally, 9% of players described SRC as: *‘A hit to the head that leads to loss of consciousness.’* Even though most of the players had some understanding or could identify signs and symptoms, there is still a misconception that SRC involves a loss of consciousness. These results indicate a knowledge gap regarding signs and symptoms.

Underreporting of an athlete’s or teammate’s suspected SRC can also be related to the stigma of athletic identity.^12^ Research in high school and college players found up to 40% of these population groups did not report injuries. In this study, players (70 %) responded that they would report a teammate’s SRC to a coach who would be able to assist with the injury. Although players had a belief in their coaches, the responses of coaches were that they did not feel empowered with the appropriate knowledge to deal with these types of injuries. These results highlight the need for improved SRC education in hockey.

### Knowledge and attitudes of hockey coaches, umpires, and official towards concussion

The CKI and CAI scores among the coaches, umpires, and officials indicate that concussion knowledge and concussion attitudes was above average. The coaches, umpires, and officials felt that they did not have the appropriate knowledge with regard to concussion, which is concerning as they have a direct involvement with the athlete. The SRC education programmes implemented in rugby, which are aimed at umpires and coaches, have shown a decrease in severe injuries and improvement in injury prevention behaviours within rugby. ^[10, 17, 18, 19]^ These results indicate a gap in SRC education of coaches, umpires, and officials.

### Limitations

Data collection occurred during the COVID-19 pandemic in South Africa, which affected response rates from participants.

Hockey players may have had survey fatigue during the period of data collection as they were required to fill in two to three Google Form questionnaires per week, negatively impacting data collection. Due to increased amounts of online research with online questionnaires during the COVID-19, pandemic response rates were lower compared to pre-COVID-19 levels. ^[20]^

The questionnaire was a self-report as opposed to a surveillance study which may not have realised extent of injuries. Also, participants may not have been completely honest in answering the questions. More players participated compared to coaches, umpires, and officials, which may not allow for a representative sample of this population group.

### Recommendations

The following recommendations are made based of the results from this study:

1. Further research into the prevalence of SRC in amateur hockey is required, including the collection of baseline data.
2. Mandatory SRC education training of hockey coaches and umpires at all levels within the SGHA.
3. Future research should investigate incidence rates per 1000 hours in order to compare SRC in hockey with other sports.

This study’s results show a need for SRC education in hockey. Improved education has decreased the number of unreported incidents and allows for the correct management of these injuries. ^[10, 19]^ The introduction of SRC protocols in other sports (such as rugby) has been shown to decrease injuries and improve athlete safety. ^[10, 19]^ Although hockey is defined as a non-contact sport, SRC incidences need to be taken seriously for the best interests of the players’ health. Sports-related concussion can appear to be an invisible injury; however, the importance and long-term health complications should not be underestimated.

## Conclusion

Overall, this study found that there was a self-reported SRC prevalence of 19% between March 2018 and March 2022. However, the low rates of SRC compared to the high rates of upper body injury incidents suggest many were not detected or reported during this period, which correlates with the high numbers of SRC that are underreported. These findings indicate that there are flaws in the management of on-field injuries which should be improved.

Although, concussion attitudes were above average for all participants, the responses from the focus group’s discussion and interviews indicated that the concussion knowledge of coaches, umpires, and officials was lacking. Despite the results that the players’ concussion knowledge was above average, they still displayed unsafe behaviour by continuing to play with a suspected SRC. Overall, these results indicate flaws in the injury management system and a gap in the concussion knowledge of the players, coaches, umpires, and officials involved in the SGHA.

## Figures and Tables

**Table 1:** Participant demographics (n=119)

	Participants	n	Mean	Median	Std deviation
**Age (years)**	Coaches, umpires, and technical official	18	38.9	35.5	10.7
	Players	101	24.5	23.0	5.4

**Starting age (years)**	Umpires	13	27.5	20.0	11.6
	Coaches	4	21.5	19.0	5.7
	Technical official	1	45.0	45.0	—
	Players	101	8.9	9.0	3.1

**Table 2:** Sports-related concussions (SRC) witnessed by coaches and officials (March 2018–March 2022)

	n	Witnessed a SRC injury (%)	Witnessed 1–5 SRCs (%)	Witnessed 6–10 SRCs (%)
**Coaches**	4	75	100	0
**Umpires**	13	92.3	91.7	8.3
**Technical officials**	1	100	100	0

**Table 3:** Sports-related concussions experienced by players between March 2018–March 2022 and before March 2018 (n=101)

	March 2018 – March 2022			Before March 2018		

	Total (%)	Male (%)	Female (%)	Total (%)	Male (%)	Female (%)
**Yes**	18.8	13.1	27.5	35.6	37.7	32.5
**No**	81.2	86.9	72.5	56.4	50.8	65
**Not sure**	0	0	0	7.9	11.5	2.5

**Table 4:** Reliability of the Concussion Attitude Index (CAI)

Factor name	Number of items	Cronbach’s alpha	Inter-item correlations mean
1st order - factor 1	4	0.74	
1st order - factor 2	3	0.81	
1st order - factor 3	3	0.61*	0.36*
2nd order factor 1	10	0.80	
Theoretical CAI	15	0.78	

* indicates reliability (>0.07)

**Table 5:** Concussion Knowledge Index (CKI) and Concussion Attitude Index (CAI) scores of participants

	n		Mean		Median		Standard deviation	

	Players	Coaches and officials	Players	Coaches and officials	Players	Coaches and officials	Players	Coaches and officials
CKI	101	18	18.9 (76%)	21 (84%)	19	22	2.6	1.9
CAI	101	18	64.6 (86%)	63.1 (84%)	66	63	7.1	4.9

CKI is a score out of 25, CAI is a score out of 75.
